# Transdiagnostic symptom dynamics during psychotherapy

**DOI:** 10.1038/s41598-022-14901-8

**Published:** 2022-06-27

**Authors:** C. O’Driscoll, S. Epskamp, E. I. Fried, R. Saunders, A. Cardoso, J. Stott, J. Wheatley, M. Cirkovic, S. A. Naqvi, J. E. J. Buckman, S. Pilling

**Affiliations:** 1 Centre for Outcomes Research and Effectiveness (CORE), Research Department of Clinical, Educational, and Health Psychology, UCL, London, UK; 2 Centre for Urban Mental Health, University of Amsterdam, Amsterdam, The Netherlands; 3 Department of Psychology, University of Amsterdam, Amsterdam, The Netherlands; 4 Department of Clinical Psychology, Leiden University, Leiden, The Netherlands; 5 North East London NHS Foundation Trust (NELFT), London, UK; 6 ADAPT Lab, Research Department of Clinical, Educational, and Health Psychology, UCL, London, UK; 7 Talk Changes: City and Hackney IAPT Service, Homerton University Hospital NHS Foundation Trust, London, UK; 8 Barking and Dagenham and Havering IAPT Services – North East London Foundation Trust, Essex, UK; 9 iCope - Camden and Islington Psychological Therapies Services - Camden and, Islington NHS Foundation Trust, London, UK; 10 Camden and Islington NHS Foundation Trust, London, UK

**Keywords:** Health care, Signs and symptoms

## Abstract

Psychotherapy is an effective treatment for many common mental health problems, but the mechanisms of action and processes of change are unclear, perhaps driven by the focus on a single diagnosis which does not reflect the heterogeneous symptom experiences of many patients. The objective of this study was to better understand therapeutic change, by illustrating how symptoms evolve and interact during psychotherapy. Data from 113,608 patients from psychological therapy services who completed depression and anxiety symptom measures across three to six therapy sessions were analysed. A panel graphical vector-autoregression model was estimated in a model development sample (N = 68,165) and generalizability was tested in a confirmatory model, fitted to a separate (hold-out) sample of patients (N = 45,443). The model displayed an excellent fit and replicated in the confirmatory holdout sample. First, we found that nearly all symptoms were statistically related to each other (i.e. dense connectivity), indicating that no one symptom or association drives change. Second, the structure of symptom interrelations which emerged did not change across sessions. These findings provide a dynamic view of the process of symptom change during psychotherapy and give rise to several causal hypotheses relating to structure, mechanism, and process.

## Introduction

Psychotherapies are effective treatments for a broad range of common mental health problems, but do not work for a substantial proportion of patients^1^. It is still not clear how therapies work, or what the processes of change occur during psychotherapies^2^. This lack of knowledge is stifling the development of novel interventions that target the putative mechanisms maintaining the disorders, hence limiting the potential for improvements in patient outcomes. Dismantling studies and trials of individual treatment components can be informative, but have frequently been hampered by low-quality methodology^3^. It is widely believed that different psychotherapies share several common causal mechanisms and operate in ways that are more similar than they are different^4–7^. Common factors, such as therapeutic alliance, are unlikely to improve our understanding of change^2^ and there is a need to focus on other mechanisms of change in psychotherapy^8,9^.

The development of psychotherapy treatments has, for the most part, been tied to specific diagnoses, yet this does not reflect clinical reality where co-morbidity is the rule rather than the exception^10^. The evidence-base for the effectiveness of psychotherapies comes from randomised control trials (RCTs) which largely focus on specific diagnoses^11^. This assumes that treatments either target an underlying disease or target a specific set of symptoms commensurate with the diagnosis. In clinical practice, co-morbidity can present challenges for clinicians in selecting the most appropriate disorder-specific treatment, or the ordering of interventions to tackle the seemingly disparate presenting problems; this can lead to some comorbid presentations being labelled as ‘complex’. The presence of comorbidity is likely an artefact of the classification of disorders^12,13^ hence significant comorbidity exists within RCTs for depression^14^ and is associated with treatment prognosis. While there are distinct features of diagnostic disorders, they are not discrete^15^, with considerable symptom overlap across disorders^16,17^. Mapping symptoms across disorders reveals how this overlap can inform an understanding of the emergence of co-morbidity^18^. Symptom heterogeneity also occurs *within* disorders^19,20^, with potential both for variability in diagnosis, or reaching the same diagnosis without any symptom overlap^21^. A transdiagnostic approach to psychopathology (i.e. aiming to identify overarching processes, by addressing causality and mechanism), might help overcome these obstacles and facilitate the identification of processes of change^22^.

Modelling change (in symptoms for example) is a fundamental means by which we can better understand mental health problems and their treatment. Methodological approaches to understanding the processes of change during psychotherapies have largely considered symptomatic change as a shift in a latent construct (e.g. a difference in the sum score on a measure of depressive symptoms, as signalling a change in the latent construct of depression). This fits with the diagnostically congruent ‘common cause’ theoretical framework, which purports that symptoms are passive and interchangeable indicators of an underlying latent disease. Systems theory and network approaches^23^ offer an alternative viewpoint, proposing that the disorder is an emergent property and that symptoms are autonomous causal agents^24,25^. According to systems theory, causal interactions between symptoms and relevant external factors can give rise to emergent states of psychopathology. An external event (e.g. loss of job) or internal event (e.g. brain trauma), can activate the system (psychological, social and biological processes), and the system will respond (e.g. symptoms activating neighbouring symptoms). While individuals may exist in states of equilibrium, crossing certain thresholds may shift the system into different self-sustaining (attractor) states, so the system may be maintained through causal loops despite the absence of the initial stressor^25,26^. If the psychotherapy activates change in an individual’s distress, this change can be observed in transition between states. During a transition, the organization of the system and facilitators of change become apparent^27^. The system may reorganise and develop as the symptoms interact or may reach a critical point and transition into a different state (i.e. ‘disordered’ to ‘healthy’). While change can be gradual or sudden, it requires significant disturbance to shift the system out of a pathological attractor state^28^.

Investigations of the processes of change in large psychotherapy samples have typically focused on identifying profiles^29^ of patients with differential outcomes and predictors of differential response trajectories^30^. Studies that have focused on symptoms have been hampered by the primary use of cross-sectional data where bidirectionality and statistical equivalence make inferences difficult^31^. Cross-sectional data limits interpretation because they cannot provide evidence for directed relationships (over time); and in cross-sectional data, statistical equivalence (i.e. having multiple models that can fit the data identically), is a larger concern than in temporal data^31^. Psychotherapy research has historically focused on between-person differences, either by comparing groups, or by studying correlations between individual characteristics^32^. Such relationships derived from group level analyses may not generalize to individuals^33^. To address these limitations, we will adopt a transdiagnostic, symptom level analysis, focusing on symptoms common across disorders, modelling change over time in a naturalistic setting of patients receiving psychotherapy. The modelling approach will also distinguish within-person variability (within a person over time and contexts), from between-person variability (stable traits and variations across persons)^34^. Within this analysis, the term within person does not refer to true within person observations (i.e. of an individual person), but of the within-person relationship of an average person (aggregated over people)^35^.

This study will focus on the dynamics between common mental health symptoms during psychotherapy. We consider the modelled symptoms to represent an important part of a broader system and consider the interactive change and self-organisation of these signs and symptoms during the process of psychotherapy. The aim is to model transdiagnostic change, across therapies and across disorders, where the symptoms modelled reflect the core symptoms of depression and generalised anxiety in the DSM-5^36^. These are dimensionally normative symptoms (e.g. tiredness, nervousness) and are diagnostic features of many disorders (Table 1). Some of these symptoms may be descriptively transdiagnostic while others could be considered mechanistically transdiagnostic^37^. Through identifying dynamics of symptom change during psychotherapy, the results can inform theory on the structure of psychopathology and functional processes of change.

**Table 1:** Symptoms captured by the PHQ-9 and GAD-7 mapped onto features of DSM-5 disorders.

Symptoms	Generalised Anxiety	Depressive disorders	Schizophrenia	PSTD	Personality disorder borderline	Bipolar	Anorexia	OCD	Insomnia	Panic disorders	Specific phobia	Social Anxiety	
Feeling nervous, anxious or on edge	**○**		**○**	**○**	**○**	**○**	**○**	**○**		**○**	**○**	**○**	10
Not being able to stop or control worrying	**○**				**○**			**○**					3
Worrying too much about different things	**○**				**○**								2
Trouble relaxing	**○**		**○**	**○**		**○**				**○**			5
Restlessness	**○**	**○**	**○**	**○**		**○**							5
Becoming easily annoyed or irritable	**○**		**○**	**○**	**○**	**○**	**○**		**○**				7
Apprehensive expectation	**○**			**○**				**○**		**○**	**○**	**○**	6
Anhedonia		**○**	**○**	**○**				**○**					4
Feeling down, depressed, or hopeless		**○**	**○**		**○**		**○**		**○**				5
Sleep difficulties	**○**	**○**	**○**	**○**		**○**	**○**		**○**				7
Feeling tired or having little energy	**○**	**○**	**○**				**○**		**○**				5
Poor appetite or overeating		**○**	**○**				**○**						3
Feeling bad about yourself/ failure		**○**		**○**	**○**		**○**						4
Concentration	**○**	**○**	**○**	**○**		**○**			**○**				6
Psychomotor retardation/agitation	**○**	**○**	**○**	**○**		**○**							5
Suicidal ideation		**○**	**○**	**○**	**○**	**○**	**○**	**○**					7
Total	11	10	12	11	7	7	7	4	4	3	2	2	

## Results

### Sample characteristics

The characteristics of the training and holdout samples are shown in Table 2. In total, combined they included 113,608 patients who attended at least three sessions. Ages ranged from 17 to 94 years old. Proportionally, there were more females (67%); and more patients from White ethnicity groups (63%). Ethnicity was reflective of the population estimates for London^38^. Most patients received High Intensity Cognitive Behavioural Therapy (CBT), and the next most frequently delivered was Low Intensity CBT, with 11% receiving a different mode of therapy [i.e., Counselling, Behavioural Couples Therapy, Dynamic Interpersonal Therapy, Eye Movement Desensitization and Reprocessing (EMDR), Mindfulness-Based Cognitive Therapy (MBCT), or Interpersonal Psychotherapy (IPT)].

**Table 2:** Sample characteristics.

	Training set		Hold out set	
	n = 67,048		n = 44,645	
PHQ-9 total: mean (SD)	14.13 (6.26)		14.15 (6.25)	
GAD-7 total: mean (SD)	12.98 (5.19)		12.98 (5.19)	
Number of sessions: mean (SD)	8.07 (4.84)		8.05 (4.85)	
Age: mean (SD)	37.76 (13.44)		37.78 (13.48)	
**Gender**				
Male	21,396	31.91%	14,466	32.40%
Female	45,357	67.65%	29,983	67.16%
Missing/not disclosed	295	0.44%	196	0.44%
**Ethnicity (ONS)**				
Asian	7024	10.48%	4681	10.48%
Black	7331	10.93%	5030	11.27%
Chinese	471	0.70%	316	0.71%
Mixed	3890	5.80%	2661	5.96%
Other	2641	3.94%	1725	3.86%
White	42,438	63.29%	28,210	63.19%
Missing	3253	4.85%	2022	4.53%
**Intervention type**				
LI CBT	25,819	38.51%	17,223	38.58%
LI Other	3815	5.69%	2502	5.60%
HI CBT	28,332	42.26%	18,909	42.35%
HI Other*	7401	11.04%	4916	11.01%
Missing	1681	2.51%	1095	2.45%
**Presenting problem (primary diagnosis)**				
Adjustment disorder	518	0.77%	333	0.75%
Agoraphobia	322	0.48%	234	0.52%
Alcohol related disorder	30	0.04%	19	0.04%
Bereavement	333	0.50%	213	0.48%
Bipolar affective disorder	39	0.06%	29	0.06%
Body dysmorphic disorder	11	0.02%	9	0.02%
Depressive episode	23,555	35.13%	15,450	34.61%
Eating disorder	156	0.23%	98	0.22%
GAD	11,041	16.47%	7232	16.20%
Hypochondriacal disorder	440	0.66%	304	0.68%
Insomnia	144	0.21%	69	0.15%
Mixed anxiety and depression	4323	6.45%	3001	6.72%
OCD	1342	2.00%	900	2.02%
Panic disorder	2402	3.58%	1647	3.69%
Personality disorders	7	0.01%	2	0.00%
PTSD	2159	3.22%	1365	3.06%
Recurrent depression	4377	6.53%	2960	6.63%
Social phobia	1994	2.97%	1410	3.16%
Somatoform disorder	335	0.50%	232	0.52%
Specific phobia	667	0.99%	427	0.96%
Unspecified anxiety disorder	548	0.82%	366	0.82%
Missing (not specified)	13,313	19.85%	8345	18.69%

*(Counselling, IPT, Psychodynamic, MBCT, EMDR).

**Figure 1:**
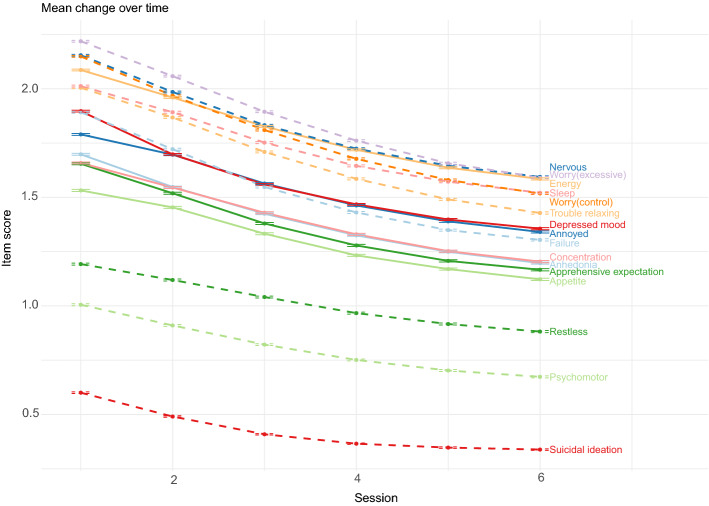
Mean symptoms scores (and standard error) across the six time points. Dashed lines are PHQ-9 items and solid lines are GAD-7 items.

There was a broad range of presenting problems (also referred to as a 'problem descriptor' in IAPT) which are diagnoses based on ICD-10 and represent the focus of treatment agreed between a patient and clinician. Figure 1 shows the mean symptoms scores across the six time points. All symptoms are shown to change over time (all p < 0.001) and the slope of the trajectory was similar across items. Mean PHQ-9 total reduced from 14.13 (SD = 6.26) at timepoint one to 10.7 (SD = 6.59) at timepoint six, and GAD-7 total from 12.98 (SD = 5.19) to 9.88 (SD = 5.71).

### Panel graphical VAR modelling

**Figure 2:**
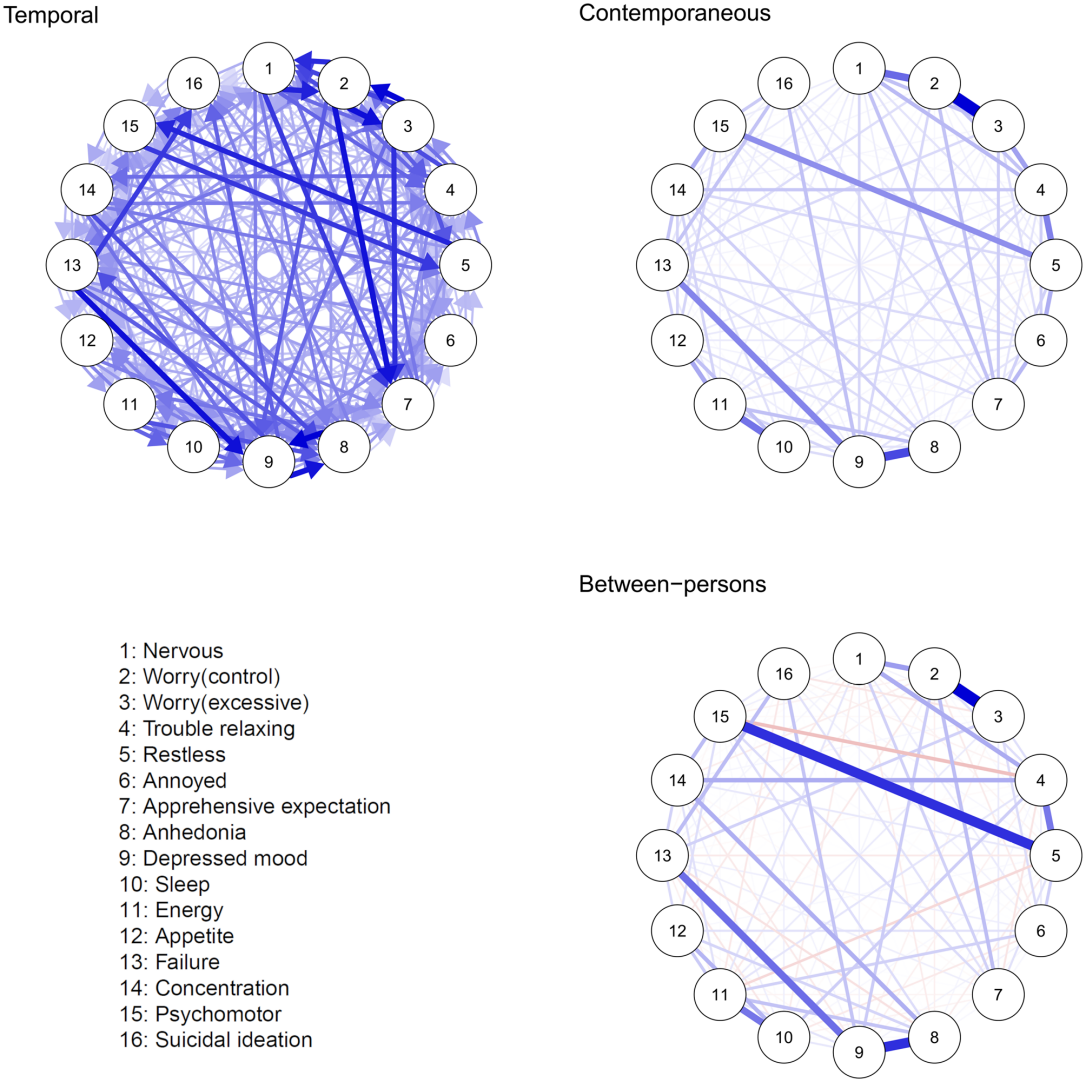
Panel graphical VAR model. Circles represent symptoms, and connections (undirected drawn as lines or directed drawn as an arrow) indicate predictive relationships. Blue lines indicate positive relationships, red lines indicate negative relationships. The width and saturation of a line indicates the strength of the relationship. In the temporal network (left), directed lines indicate where a symptom predicts another symptom at the next session after controlling for all other variables. Within the contemporaneous (middle) lines represent partial correlations between symptoms at the same timepoint, after controlling for all other variables and temporal effects. The between-persons network (right) indicating partial correlations between stable averages. We only plot significant edges, and the visualisation of autocorrelations in the temporal network has been omitted to improve visualisation; a figure including autocorrelations can be found in supplementary materials.

The panel graphical VAR was estimated in the training sample. Confidence interval (CI) plots for the model are available at https://osf.io/gp6dw/. Figure 2 shows the temporal, contemporaneous, and between-person networks. The training model (n = 68,165) contained 16 items and six timepoints, resulting in 544 estimated parameters. The fit statistics for all models are displayed in Table 3. The root-mean-square error of approximation (RMSEA) of the training model was 0.014 (95% CI 0.014; 0.015), and incremental fit indices were excellent. The confirmatory model in the holdout sample (n = 45,443) showed excellent fit. This was further supported by the equality constrained model which also showed excellent fit. Finally, the nondetrended model showed good fit, with the high to near perfect spearman correlations between detrended and nondetrended adjacency matrices (temporal r = 0.82; contemporaneous r = 0.99; between r = 0.93).

**Table 3:** Fit statistics across panel graphical VAR models.

	Training model	Hold out model	Confirmatory model	Equality constrained model	Training model (nondetrended)
df	4208	4208	4464	8672	4208
Chisq	63,960	48,365	25,525	108,247	119,256
NFI	0.984	0.982	0.982	0.984	0.970
PNFI	0.908	0.906	0.906	0.935	0.895
TLI	0.984	0.982	0.984	0.984	0.969
RFI	0.983	0.980	0.980	0.983	0.968
IFI	0.985	0.983	0.985	0.985	0.971
CFI	0.985	0.983	0.985	0.985	0.971
RMSEA	0.014	0.015	0.017	0.014	0.020
RMSEA 95% CIs	0.014:0.015	0.015:0.015	0.017:0.017	0.014:0.014	0.020:0.020

### Temporal network

The saturated temporal network model was dense, with mostly small but significant parameters at the adjusted p-value (p < 0.0001), mean association: r = 0.03 (median = 0.02). The network displayed moderate to large autocorrelations, with suicidal ideation as the strongest (r = 0.27).

The most prominent bidirectional associations, following the autocorrelations, were between depressed mood and anhedonia; excessive worry and difficulty controlling worry; difficulty controlling worry and feeling nervous/anxious; psychomotor retardation/agitation and restlessness (all |r| > 0.05). The most prominent unidirectional associations (|r| > 0.05) were between depressed mood to feeling like a failure; apprehensive expectation to difficulty controlling worry; apprehensive expectation to excessive worry; apprehensive expectation to feeling nervous/anxious and suicidal ideation to feeling like a failure. While these associations are the most prominent, the cut-off of 0.05 was arbitrary and 34 associations had CIs in the range of 0.05.

Considering the centrality of the items, depressed mood (z = 2.37), anhedonia (z = 1.74), feeling nervous (z = 0.96) and apprehensive expectation (z = 0.94) had the strongest In-Expected Influence (all z > 0.9). Excessive worry (z = 1.32), difficulty controlling worry (z = 1.22) and trouble relaxing (z = 1.10 > 1), has the strongest Out-Expected Influence.

### Contemporaneous and between person models

The within-person contemporaneous network was also dense with most associations highly significant (p < 0.0001). Contemporaneous within/between networks were highly correlated (r = 0.79), suggesting a high degree of homogeneity of effects—i.e., low between-person differences. The strongest associations within-persons (|r| > 0.2) were excessive worry and difficulty controlling worry; depressed mood and anhedonia; difficulty controlling worry and feeling nervous; energy and sleep; restlessness and trouble relaxing; and feeling like a failure and depressed mood. At the within-persons level, the most central items were difficulty controlling worry, depressed mood, trouble relaxing and excessive worry (z > 1). The strongest associations between-persons (|r| > 0.3) were excessive worry and difficulty controlling worry; depressed mood and anhedonia; psychomotor agitation/retardation and restlessness; anhedonia and depressed mood; depressed mood and feeling like a failure; and restlessness and trouble relaxing. In the between-persons network, depressed mood, and difficulty controlling worry were most central (z > 1). Centrality of items between the within and between networks was correlated r = 0.72.

Unregularised, cross-sectional networks using the Gaussian graphical stepwise model selection (“ggModSelect”) algorithm^35^ at each time point (see https://osf.io/gp6dw/), could not be distinguished from unity (r = 0.99), indicating near perfect replication of network structures across all six timepoints. The mean density of networks (the sum of all edges within each network) was 7.27 (sd = 0.09, range: 7.12 to 7.34), incrementally increasing over time.

## Discussion

This study explored dynamics of symptom change during psychotherapy. Using sessional symptom data from a large sample receiving treatment for common mental disorders, the results show a large co-occurrence of symptom change over time. Symptoms decreased across the board, and there was a strong temporal dependence between various symptoms. The network structure of associations, however, remained the same. Mean changes were somewhat different for different symptoms, but they tended to change together. The results of these analyses are statistically reliable; generalise to a holdout sample; and provide insights in the temporal effects and whether these associations covary at the trait (between person), or state level (within person). While we do not know whether findings generalize to a non-psychotherapy (e.g. waitlist control) condition, we assume that some of the observed patterns in the data are due to psychotherapy, e.g. the overall symptom reduction over time. In the rest of the discussion, we consider interpretation, implications, specific findings, strengths and limitations.

Interpretation of the dynamics can be viewed in several ways. These findings highlight a syntactical equivalence^39^, with the results supporting both common cause and systems theory which are often considered to be diametrically opposed. A dynamic systems theory view is that a broad range of symptoms were active and that these in turn influenced other symptoms over time. From a common cause perspective, the density of the network would suggest a common latent variable (i.e. symptoms reflect an unobserved construct). We might infer causal (temporal) associations between symptoms from the temporal network model, supporting a systems interpretation, as a common cause model assumes no direct causal relations between observations. It is also likely that both are true simultaneously; a hybrid model where the common cause reflects onset and the dynamic system, maintenance^40^. These theories are under determined given the data, requiring experimental intervention to differentiate these theories^31^. From a systems perspective, one might have expected a sparser model with bridging symptoms identified between clusters of similar symptoms to explain the development of co-morbidity^41^. The sparsity usually revealed in network studies may be related to a combination of using underpowered small datasets and regularization. Therefore, in this large sample, the density of significant associations may be more representative of the actual complexity of the psychopathological system (i.e. closer to the true model^42^) and as such the sparsity assumption may be invalid.

This brings us to the clinical implications. The models using detrended and undetrended data were comparable which strengthens the ability to make inferences around processes of change^43^. Change mechanisms or causal effects were also most likely to be identified within the contemporaneous network where confounding by stable variables was mitigated^44^. There is heterogeneity in the sample, with variation in individual factors, diagnoses, and types of therapy received. On the one hand, if there is a common process underlying all psychotherapeutic approaches (e.g. exposure^5^), certain symptoms may respond similarly (regardless of sample heterogeneity), where symptoms are interchangeable indicators of a process (e.g. avoidance). Change in one symptom should result in changes throughout the network leading to a change in overall symptom severity. Such an interpretation rests on assumptions that: there are no other external effects; that we have modelled all relevant variables; and that we have the accurate time-steps by which variables evolve. On the other hand, certain symptoms were more dominant, by which we mean they had stronger and more numerous edges, within the network structures. If such symptoms exhibited the highest causal force, which is one possible interpretation of the data, then these symptoms may have triggered changes that rippled through the network. If this were the case, worry (excessiveness and controllability), along with trouble relaxing, appeared to hold the strongest influence on other symptoms. The influence of these symptoms and depressed mood were also highlighted at the contemporaneous level. Feeling nervous, depressed mood and anhedonia were most influenced by other symptoms. This may suggest that during psychotherapy, on average, strategies targeting worry and trouble relaxing may bring about changes throughout the network, either directly or through pathways to the most influenced symptoms.

Some specific findings are worth discussing in some detail. The association between depressed mood and anhedonia was consistent with other findings^45^, representing core symptoms of depression^46^, and excessive and uncontrollable worry as a transdiagnostic process (i.e. repetitive negative thinking)^47,48^. Worry symptoms (excessiveness/controllability) covaried, as did depressed mood and anhedonia. The controllability of worry covaried with feeling nervous; energy with sleep; restlessness with trouble relaxing; and feelings of failure with depressed mood. Suicidal ideation at one session, predicted by itself at an earlier session, was the strongest association in the temporal network, with suicidal ideation predicting a sense of failure and depressed mood at the next session. To a lesser degree, depressed mood also influenced suicidal ideation at the next timepoint. This is notable given that suicidal ideation is generally a peripheral symptom in many network studies^49^ and whilst considered clinically important, it is rarely targeted with direct interventions in the same way as depressed mood or worry. The emergence of this association may be due to the use of a considerably larger and naturalistically treated patient sample compared to most prior studies which may have encountered floor effects on measures of suicidal ideation given their smaller and often non-clinical samples.

According to dynamic systems theory, differences between states can reflect attempts for correction and, depending on the mechanism of change, can lead to a re-organisation in the system—shift in state (e.g. from ‘disordered’ to ‘healthy’). Within this panel model, the structure of the network does not change over time, across the time-period measured (six sessions). A critical tipping point may not have been reached (i.e. on average, the ‘disordered’ state is maintained). On average, the most change occurred within this timeframe, but changes to the stability and density of the networks may have altered were further sessions included. Such shifts may be revealed in separate dynamic networks of those whose symptoms remit versus those whose symptoms persist, or more notably at the idiographic level, where identifying these state transitions could have a deterministic effect on treatment outcome.

The study captured between session changes and appeared, to a degree, to have also captured changes that occurred at shorter intervals (the contemporaneous network displayed symptom dynamics which unfolded faster than the timeframe of measurement^40^). The measurement approach may not have allowed for sufficient granularity of symptomatic change processes during psychotherapy. These may be better captured by more frequent measurement, including approaches less reliant on retrospective recall, such as the use of ecological momentary assessments (EMA). The implementation of EMA during therapy might allow for idiographic modelling of change processes which could directly inform the therapeutic process as it unfolds^50^.

A clear strength of this study lies in using routine clinical data from a large sample in a naturalistic setting and not constraining analyses to any diagnostic category or specific therapy type. Findings may therefore be generalizable to a broad population of adults seeking psychotherapies for common mental disorders. There are limits to the generalizability as only services in the London area were included and all participants received healthcare free at the point of use, so replication in other settings and locations may be required. The study captures provisional diagnosis; these are not formal diagnosis and there was no assessment of co-morbidity.

There are limitations to deriving true causal relationships between symptoms in this study. This requires consideration the assumptions of the statistical model. First, while we can identify temporal precedence, and the approach allows for conditional inferences across levels^51^, the modelling approach captured group level processes. Second, the model does not capture measurement error, and cannot account for the absence of unmeasured external variables (other core psychopathological symptoms) or time-varying confounders. Third, some of these associations may be due to topological overlap (although we tested for this and it was not present across the cross-sectional networks). Fourth, ergodicity, while not a required assumption for causal statements, is implausible in in such a heterogeneous sample, although the average group model was highly similar to the average individual model over time. Finally, the model does not reveal likely subgroups with different trajectories of change. Indeed, in a similar sample, four trajectories of change based on the PHQ-9 and GAD-7 sum scores were identified^29^. As such, the findings from this study can only be taken as potential causal associations and inferences about intra-individual processes of change during psychotherapy in this sample relate to a hypothetical average person. Identification of subgroups combined with latent growth network modelling^52^ may offer additional insights into the change mechanisms during psychotherapy, help us understand how different subgroups respond to therapy, and what specific factors may contribute to better outcomes.

This study mapped the symptom dynamics during psychotherapy but there is still a question about what is influencing this change. It is uncertain as to whether these changes occur due to: in-session process such as exposure, as a transdiagnostic procedure^5^; features of the therapeutic alliance such as the development of epistemic trust^53^; therapeutic procedures such as developing strategies to address repetitive negative thinking^54^; between session behaviour change^55^; or regression to the mean^56^. Such features of psychotherapy, where measurable could be integrated into moderated network models where the network is conditioned on the mechanism of change^57^. Such advances will help us to further understand how psychotherapy works.

The focus on symptoms feeds into a biomedical understanding of mental health difficulties and omits many important variables (e.g. experiential and quality of life related constructs). A more comprehensive biopsychosocial model would require including markers generally associated with prognoses regardless of the type of treatment received including markers of the severity of the mental health condition, and also social support, life events, sociodemographics and socioeconomic factors^58–61^. While this introduces complexity at the modelling and data collection levels, developments across both these areas will further develop our understanding of change during therapy. Nonetheless, this study may help elucidate the biobehavioural understanding of change during psychotherapy.

Much of the research to date has focused on change at the mechanistic level or sum-score changes, with little focus on change at the level of specific symptoms and even less on their temporal associations. The relationship between symptoms can help to predict outcomes and potentially inform the development of more targeted treatments^11,62^. It may also help inform an understanding of phenomena such as early sudden gains^63^. This study provides a significant contribution to the network literature: informing network methodology; addressing concerns about network replicability^64^; and overcoming barriers present in previous research limited by sample size and cross-sectional design.

## Methods

All methods were carried out in accordance with the Health Research Authority guidelines. NHS ethical approval was not required for this study (confirmed by the Health Research Authority July 2020, reference number 81/81). The data were provided by the IAPT services for evaluation as part of a wider service improvement project conducted in accordance with the procedures of the host institution and the NHS Trusts which operate the IAPT services (project reference: 00519-IAPT). At their initial contact with services all patients are informed that their data are sent to NHS Digital as part of national reporting, and may be used for research and service improvement by the services, and they are given the option to opt out of this if they wish. Only anonymised data from those patients that were considered to have opted-in for their data to flow in this way were included in the current project. No patient identifiable data were available to the research team.

### Participants

We analyzed data from patients that received psychological therapy from eight Improving Access to Psychological Therapies (IAPT) services in the North and Central East London IAPT Service Improvement and Research Network (NCEL IAPT SIRN)^65^ IAPT services provide evidence-based psychological treatments for common mental health disorders and are mandated to collect outcome measures at each session, which has resulted in over 98% pre-post treatment data availability^66^. In IAPT, high intensity therapy includes CBT, Behavioural Activation, Counselling, Interpersonal Psychotherapy, Short-term Psychodynamic Psychotherapy and EMDR, typically weekly for 10 to 16 session lasting 50 to 60 min, while low intensity therapy, tends to involve four to eight sessions of 30 min with practitioners guiding patients in the use of self-help material or computerized programmes based on CBT or Behavioural Activation^67^.

For this study, patients were included if they received a minimum of three psychological therapy treatment sessions and if data were available on all the individual symptom items from the requisite symptom measures (detailed below). Only data from the first six treatment sessions were analysed regardless of the total number of sessions a patient received if beyond six.

### Measures

Each session patients completed: the Patient Health Questionnaire 9-item version (PHQ-9^68^) a brief measure of depressive symptoms; and the Generalized Anxiety Disorder Scale 7-item version (GAD-7^69^), a measure of generalised anxiety disorder symptoms.

### Plan of analysis

The analyses involved estimating a panel graphical vector-autoregressive model (panel GVAR) in the training data and confirmatory models to test generalizability by: (a) fitting the network model in the holdout sample; and (b) testing for parameter invariance between datasets by implementing equality constraints (i.e. edges constrained to be equal between the training and holdout set). Finally, cross sectional networks were estimated to visualise the network structure at each timepoint.

Treatment length differs across modalities, and substantial change typically occurs early in psychotherapy^63,70^, with a previous analysis in a similar sample indicating that the trajectory of change could be identified by the third session for most patients, and by the sixth session for the remaining patients^29^. Temporal dynamics were modelled across the first six sessions, chosen to capture these early causal dynamics during this period. The cap of six was also informed by the constraints of model complexity where convergence issues arise with each additional wave.

The dataset was randomly split (60:40) into a training and holdout sample. Multilevel linear mixed-effects models, with maximum-likelihood estimation, were used to examine change across sessions for each item within the training sample. Data were detrended within each split, removing trend effects in means and variances (standardised per variable, per time-point). Whilst not an assumption of the modelling procedure, the aim was to improve model fit. This way, within-person and between person relationships between the variables of interest could be investigated after taking growth processes into account.

A lag-1 panel-GVAR using full-information maximum likelihood (FIML) estimation was fitted using the psychonetrics package^71^. As we modelled observed variables (i.e. no latent factors), the model is similar to a cross lagged panel model with random intercept with the covariance structure for the first time point implied by the temporal structure. By separating within from between person variance, the lagged relationships equal within-person variance^72^. Using maximum likelihood estimation, all edges were included in the temporal, contemporaneous, and between-subject networks. Residual variances were estimated using a Cholesky decomposition. Missingness was handled using FIML which adjusts the likelihood function so that each case contributes information on the variables that are observed. Multiple imputation and FIML will come to similar results when data are missing at random^73^.

Confirmatory testing involved fitting a model in the holdout sample, specifying the adjacency matrix. Parameter invariance between samples was tested by estimating a model where we introduced equality constraints. Finally, we estimated a non-detrended model for comparison, where models are comparable (detrended and nondetrended). This suggests that detrending has not biased results, supporting causal inference^43^.

Model fit was assessed using a series of fit statistics. To assess models, we used relative fit indices (Normed Fit Index (NFI), Tucker Lewis index (TLI), Incremental Fit Index (IFI), Parsimony-Adjusted Measures Index (PNFI) and Relative Fit Index (RFI)) which compare a chi-square for the model to one from a baseline model and noncentrality based indices (Comparative Fit Index (CFI) and Root Mean Square Error of Approximation (RMSEA) with 95% Confidence Intervals (CI). Absolute fit indices, Chi-square (χ^2^) was reported but not interpreted given it’s sensitive to sample size. Of these indices, PNFI values above approximately 0.75 and RMSEA values < 0.05 indicate good fit; for the others, values ≥ 0.90–0.95 and are variably accepted as cut-offs for good fit^74^.

The panel data model from the training dataset, along with separate temporal (within-persons temporal patterns); contemporaneous (within-person fluctuations predicting other within-person fluctuations in the same time-window, after controlling for temporal effects); and between-persons (associations between stable averages) network models, were used for visualisation and interpretation of parameters. Within the graphical model, the conditional dependence relations between symptoms are estimated, where the line between nodes, (“edges”), represent shared unique variance that may be an indication of a causal pathway, or a common external (unmeasured) cause. The centrality metric, Expected Influence (EI), was estimated within each network. EI is sum of edge weights, either to, “In EI”, or from, “Out EI”, a symptom, reflect the centrality of the symptom within the network.

We estimated unregularised Graphical Gaussian models (GGM) at each timepoint, using undetrended data, to assess the network structure across sessions. At each timepoint we assessed for topological overlap using the goldbricker function^75^. Estimations were based on the Spearman covariance matrices and following an iterative modelling procedure using the Extended Bayesian Information Criterion (EBIC). Selecting unregularised GGMs according to EBIC has been shown to converge to the true model^76,77^. The ggmModselect algorithm runs 100 graphical lasso models (estimating sparse inverse covariance matrices using a lasso (L1) penalty), refits all models without regularisation, adding and removing edges until EBIC no longer improved^78^. The best performing model (EBIC parameter) was selected to provide a conservative GGM (high specificity).

## Data Availability

All materials have been made publicly available via the Open Science Framework and can be accessed at https://osf.io/gp6dw/. Data [matrices to reproduce the models] that that support the findings of this study are also available there. The raw data are not publicly available due to them containing information that could compromise participant privacy/consent. The raw data that support the findings of this study are available on request from the corresponding author JEJB subject to appropriate permissions from the custodians of the data. restrictions apply to the availability of these data, which were used under license for the current study. The data were provided by the IAPT services for evaluation as part of a wider service improvement project conducted in accordance with the procedures of the host institution and the NHS Trusts which operate the IAPT services (project reference: 00519-IAPT).
